# ChatGPT and Artificial Intelligence in Hospital Level Research: Potential, Precautions, and Prospects

**DOI:** 10.14797/mdcvj.1290

**Published:** 2023-11-16

**Authors:** Hassaan B. Arshad, Sara A. Butt, Safi U. Khan, Zulqarnain Javed, Khurram Nasir

**Affiliations:** 1 Houston Methodist DeBakey Heart & Vascular Center, Houston, Texas, US; 2 Houston Methodist Research Institute, Houston, Texas, US

**Keywords:** medical research, artificial intelligence, ChatGPT, outcomes research, data analytics

## Abstract

Rapid advancements in artificial intelligence (AI) have revolutionized numerous sectors, including medical research. Among the various AI tools, OpenAI’s ChatGPT, a state-of-the-art language model, has demonstrated immense potential in aiding and enhancing research processes. This review explores the application of ChatGPT in medical hospital level research, focusing on its capabilities for academic writing assistance, data analytics, statistics handling, and code generation. Notably, it delves into the model’s ability to streamline tasks, support decision making, and improve patient interaction. However, the article also underscores the importance of exercising caution while dealing with sensitive healthcare data and highlights the limitations of ChatGPT, such as its potential for erroneous outputs and biases. Furthermore, the review discusses the ethical considerations that arise with AI use in health care, including data privacy, AI interpretability, and the risk of AI-induced disparities. The article culminates by envisioning the future of AI in medical research, emphasizing the need for robust regulatory frameworks and guidelines that balance the potential of AI with ethical considerations. As AI continues to evolve, it holds promising potential to augment medical research in a manner that is ethical, equitable, and patient-centric.

## Introduction

Generative Pre-trained Transformer (GPT) models have revolutionized the field of natural language processing (NLP) with their ability to understand, generate, and interact in human-like language. One such model, ChatGPT, developed by OpenAI, represents an advanced, conversational AI model based on the GPT-4 architecture.^1^ Trained on diverse internet text, it can generate coherent, contextually relevant responses and accomplish specific language tasks, making it a powerful tool with potential applications in medical research.

## Applications in Research

The capabilities of ChatGPT extend to a broad spectrum of research-oriented tasks that could be a game-changer in the medical field. Its potential areas of application encompass academic writing assistance, data analytics, statistics handling, and even code generation, each of which will be explored further in the following sections.

### Academic Writing Assistance

As medical research often entails extensive writing, ranging from literature reviews to detailed reports, ChatGPT can prove to be a valuable tool. It can assist researchers by drafting text, refining it for clarity and conciseness, generating ideas for discussion, and even suggesting relevant literature.^1^ For instance, consider a cardiologist who is studying the role of certain genetic markers in myocardial infarctions. ChatGPT could assist the researcher by generating a structured outline for a literature review based on provided keywords such as “myocardial infarction,” “genetic markers,” and “cardiovascular disease.” The AI could also suggest pivotal papers that should be included in the review based on its extensive training data.

### Data Analytics

In the age of big data, ChatGPT’s ability to aid in preliminary data exploration and understanding is invaluable. It has the capability to simplify complex datasets into readable summaries, thereby saving researchers considerable time and effort.

Take the case of a large dataset on heart disease prevalence across different demographics. Navigating this data manually would be a daunting task. Here, ChatGPT could generate a summary detailing the distribution of key variables such as age, gender, and associated risk factors. For instance, it might identify patterns such as a higher prevalence of heart disease among males over age 60 or correlations between obesity and heart disease incidence. This preliminary analysis can then guide more nuanced and detailed study.

### Statistics Handling

Statistical analysis forms the backbone of any medical research study. Here too, ChatGPT can provide guidance on appropriate statistical tests for data analysis based on the nature and distribution of the data.^2^ For instance, suppose a researcher is working with a dataset exploring the relationship between smoking and lung cancer with variables like age, smoking frequency, and cancer incidence. ChatGPT could suggest suitable statistical tests such as Chi-square tests for categorical data, t-tests for comparing means, or regression models for exploring relationships between variables. However, it is important to remember that ChatGPT is an AI model, not a statistician. Thus, it becomes essential to corroborate AI guidance with conventional statistical resources or to consult a statistician to confirm the appropriateness of the suggested tests.

### Code Writing

Writing code for data processing and analysis can be a bottleneck for researchers unfamiliar with programming languages. ChatGPT, equipped with the ability to generate syntactically correct code snippets, could ease this process considerably.

For example, a researcher tasked with calculating the mean age of patients from a CSV dataset might not know how to start with Python or R. In this scenario, ChatGPT could generate a Python code snippet using the pandas library to import the CSV file, extract the “age” column, and calculate the mean. This not only saves time but also helps those learning programming by providing practical, context-based examples.

Each of these applications of ChatGPT in medical research brings its unique benefits. By assisting in academic writing, data analysis, statistical guidance, and code generation, ChatGPT has the potential to be a game-changing tool in the hands of medical professionals and researchers. However, as with all tools, effective use requires understanding its capabilities and limitations, which is the responsibility of the user.

## Maximizing the Use of ChatGPT

For the average user, optimizing the use of ChatGPT involves a combination of writing effective prompts, leveraging the capabilities of the AI, and supplementing it with other tools and resources.

### Prompt Writing in ChatGPT

The effectiveness of ChatGPT is significantly influenced by the quality of the prompts it receives. As an AI language model, ChatGPT generates responses based on the input provided, and the clarity and specificity of the prompt play a crucial role in determining the usefulness of the generated response. Crafting well-structured and informative prompts can help elicit more relevant and accurate outputs, enhancing the utility of ChatGPT in various research contexts.

Take this example of a less effective prompt: “Tell me about high sensitivity troponins?” This prompt is vague and lacks specificity, which may lead ChatGPT to generate a broad and general response that might not be particularly helpful for a cardiology researcher or a clinical cardiologist. The AI model could produce a response encompassing various aspects of the topic without focusing on any particular aspect or providing in-depth information.

Now, take this example of a more effective prompt: “Provide a summary of the role of high-sensitivity troponin tests in the early diagnosis of acute myocardial infarction and their prognostic value especially in the setting of non-ST elevation myocardial infarction. Also compare it with conventional Troponin-I, focusing especially on research from 2018 onwards.”

**Figure 1:**
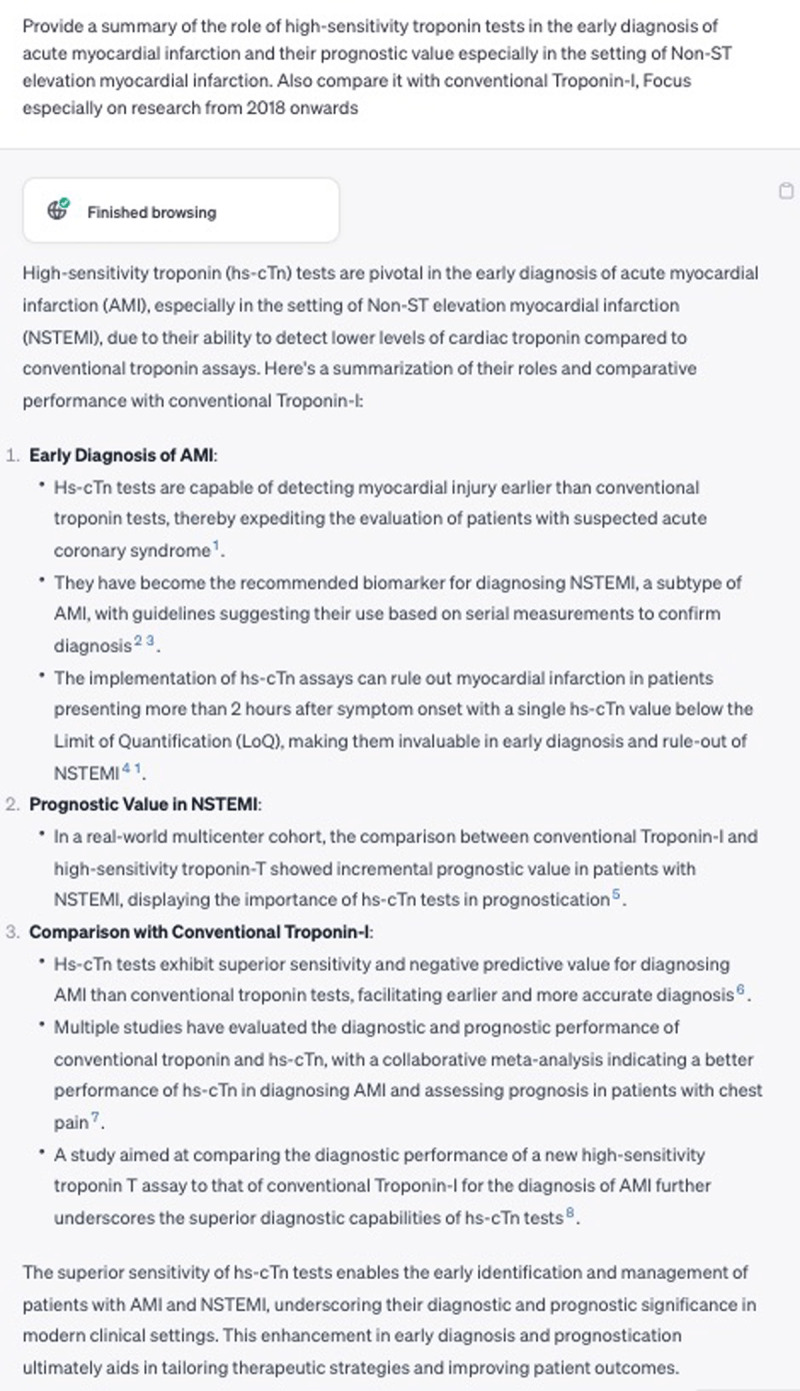
A screenshot of AI-generated response from ChatGPT based on a prompt with specific medical input.

In this case, the prompt is specific, well-defined, and clearly outlines the desired information (Figure 1). By providing a clear context and explicitly mentioning the area of interest (high-sensitivity troponin tests) and the research focus (early diagnosis of acute myocardial infarction and prognostic value), the prompt is more likely to result in a detailed, focused, and relevant response from ChatGPT.

The quality of the prompts provided to ChatGPT plays a crucial role in determining the utility of the AI model in medical research, including cardiology. By crafting clear, specific, and informative prompts, researchers can harness the potential of ChatGPT more effectively and obtain more relevant and accurate information.

### Applications of GPT-4

GPT-4 capabilities are not limited to direct interaction with the AI model via text prompts. Its sophisticated language understanding and generation abilities have been leveraged to develop a variety of innovative applications in different fields.

GPT-4 is integrated into various platforms that enhance their capabilities. Education technology platforms like Outwrite and Grammarly use it for real-time grammar and writing suggestions. Creative writing tools like ShortlyAI leverage it for story ideation and plot development. GitHub’s Copilot, a coding assistant, utilizes GPT-4 for code generation and debugging. Customer service chatbots employ it for automated responses to queries.

The Genie App extends GPT-4 functionalities by introducing multimodal capabilities. It uses AI models for image recognition, enabling initial interpretation of uploaded images, though professional validation is crucial. The app also allows document analysis, where users can upload PDFs for AI-driven summary, key finding extraction, or further research suggestions. Additionally, it offers URL summarization for quick comprehension of webpage content. These features, moving beyond traditional text-based interactions with ChatGPT, provide a versatile AI assistant experience.

## Cautions and Contemporary Issues in Using AI and ChatGPT in Medical Research

While artificial intelligence AI systems like ChatGPT hold immense potential for enhancing medical research, several contemporary issues underscore the need for caution and judicious use. These primarily revolve around data security, model reliability, biases, and the timeliness of information provided by AI.

### Data Security/Patient Confidentiality

The first major concern pertains to the handling of sensitive healthcare data. Unlike traditional statistical software that operates locally on a machine, the interactions with AI systems like ChatGPT are stored and processed on cloud servers.^3^ This raises potential issues related to data privacy and security.

Healthcare data is often sensitive and subject to stringent privacy regulations such as the Health Insurance Portability and Accountability Act (HIPAA) in the United States (US) or General Data Protection Regulation in the European Union (EU).^4^ When using AI systems, it becomes paramount to ensure that data is de-identified and confidentiality is maintained. This includes removing any personally identifiable information before processing and ensuring that any data used is securely stored and transmitted.

For institutions, implementing robust data governance policies and ensuring compliance with existing data protection regulations becomes essential. AI vendors, on the other hand, must provide transparent information about their data handling procedures and ensure they have robust security measures in place to prevent data breaches.^5^

### Reliability and Biases

Another issue is the reliability of AI outputs. Despite their sophistication, Radford and colleagues showed that AI systems can produce erroneous outputs or exhibit biases based on the data on which they were trained.^1^ For instance, if the data used to train the model had an underrepresentation of certain ethnicities, the AI might produce biased results when applied to a diverse population.^6^

This problem is not limited to ChatGPT; rather, it is a broader issue in the field of AI. Therefore, it is crucial to validate AI outputs using established methodologies and peer review to ensure their accuracy and reliability. Furthermore, efforts should be undertaken to make AI training data as diverse and representative as possible to mitigate biases.

### Timeliness of Information

Finally, the timeliness of information provided by AI systems is a significant factor. As of its last update in September 2021, ChatGPT’s knowledge base does not include any developments or studies past that date. This has important implications for medical research, where new discoveries and advancements occur on a regular basis.

The onus, therefore, lies with researchers to stay current with the latest research in their respective fields and not to rely solely on AI systems for the most recent information. Instead, AI should be used as a tool to assist in research, while traditional methods of staying updated (like reading recent journal articles) should still be employed.

### ChatGPT and Data Hallucinations

A unique challenge posed by AI language models like ChatGPT is the phenomenon of data hallucinations. In the context of AI, “hallucination” refers to the model generating information for which it was not explicitly trained and which may not be accurate or even exist in reality. For instance, ChatGPT sometimes may generate references or quotes that seem legitimate but are entirely fabricated or “hallucinated” by the AI.^1^ This can potentially mislead users who might assume the information provided by the AI is factual and backed by real sources.

This behavior stems from the model’s design and training process. ChatGPT is trained to predict the next word in a sentence based on the preceding words, and it does this by learning patterns from the large amount of text data it was trained on. However, it does not have an explicit understanding of truth or factual accuracy. It generates text that is likely based on its training data, but it cannot verify the accuracy of the information it produces.

Data hallucinations can be particularly problematic in the context of medical research, where accuracy and factual correctness are paramount. A researcher using ChatGPT might unknowingly rely on an erroneous piece of information or a nonexistent reference generated by the AI.

### AI Interpretability: Black Box

The interpretability of AI systems, particularly those based on deep learning, poses a significant challenge in the medical field. These AI models, often described as “black boxes,” can make predictions or decisions that are hard to interpret or explain. While their predictions may be accurate, the lack of transparency in their decision-making processes can be problematic, especially in the healthcare field where understanding the rationale behind a diagnosis or prediction is crucial.

For example, consider an AI model trained to predict the likelihood of a patient developing heart disease based on various factors such as age, gender, lifestyle, and genetic predisposition. While the model might accurately predict the risk, it may not provide clear insights into which factors contributed most to the prediction. Was it the patient’s age, their genetic profile, or perhaps a combination of factors? Without this insight, clinicians and patients may be hesitant to trust the AI’s prediction no matter how accurate it might be.

This lack of interpretability also poses challenges in validating AI models. If the model’s approach to making decisions is not clear, it becomes harder to identify and correct errors or biases based on those decisions.

A growing body of research focused on improving the interpretability of AI systems is becoming available, including techniques such as SHapley Additive exPlanations (SHAP) and Local Interpretable Model-Agnostic Explanations (LIME). However, much work remains before these techniques become mainstream in healthcare AI applications.^7^

### AI-Induced Disparities in Health Care

Another critical concern is the risk of AI-induced disparities in health care. AI systems are trained on data, and if this data is unrepresentative or reflects existing biases, the AI could perpetuate or even exacerbate these biases.

For example, if an AI diagnostic tool is trained predominantly on data from one ethnic group, its accuracy might be significantly lower when used on patients from other ethnic groups. A study by Obermeyer et al.^6^ found that a widely used healthcare algorithm was less likely to refer Black patients to programs that aim to improve care for patients with complex medical needs, largely because the algorithm was trained on data reflecting systemic biases in health care.

Addressing these disparities requires concerted effort at every stage of AI system development, from the collection of diverse and representative training data to the continuous monitoring and adjustment of AI systems post-deployment.

## AI Ethics and Regulations

Regulatory bodies across the globe have recognized these challenges and are working toward developing frameworks to guide AI use in health care. For instance, the US Food and Drug Administration has proposed a regulatory framework for AI-based Software as a Medical Device (SaMD), acknowledging the unique characteristics of AI/ML-based software modifications.^8^ Likewise, the EU has proposed regulations that lay down harmonized rules on AI and promote transparency, accountability, and user autonomy in AI systems.^9^

However, much work remains to be done. The dynamic nature of AI systems, which continually learn and adapt, poses challenges for traditional regulatory approaches that are designed for static products. Furthermore, while guidelines for data privacy exist, they need to be updated and adapted to the realities of AI and big data.

## The Future of AI in Medical Research

The advent of AI and chatbots like ChatGPT in medical research ushers in a new era characterized by unprecedented efficiency and precision. With the ability to streamline research processes, facilitate sophisticated data analysis, improve patient interactions, and assist in decision making, AI technologies are poised to profoundly transform the medical research landscape.^10^

The impact of AI extends far beyond language models like ChatGPT. Predictive analytics, for example, leverages AI to revolutionize epidemiological studies and disease forecasting. By analyzing patterns in vast datasets, AI can identify potential risk factors, predict disease outbreaks, and forecast the spread of diseases with impressive accuracy.^5^

AI-powered diagnostics represent another promising field. By training on thousands of patient images or scans, AI can learn to identify diseases, such as cancer or pneumonia, often with accuracy comparable to or even exceeding that of human experts.^11^ For example, Google’s DeepMind has demonstrated impressive results in diagnosing eye diseases from retinal scans, while IBM’s Watson has been used for personalized treatment recommendations in oncology.

AI also has the potential to improve patient care and interaction. For instance, chatbots can be used for initial patient triage, answering common queries and even monitoring patient health, freeing healthcare professionals to focus on more complex tasks.^12^

## Conclusion

In summary, the introduction of AI and chatbots like ChatGPT in the medical research domain holds exciting potential. They are useful tools for academic writing, data analysis, statistics handling, and code generation. However, the use of these technologies requires a nuanced understanding of their limitations, particularly concerning data security and the risk of bias or error in outputs. As we move into the future, we must strive for a balance between leveraging AI’s potential and ensuring ethical considerations are at the forefront of this technological revolution.

As we navigate this promising yet challenging terrain, collaboration between AI specialists, medical researchers, ethicists, and policymakers will be paramount. Through such interdisciplinary cooperation, we can work towards a future where AI not only augments medical research but does so in a way that is safe, ethical, and beneficial for all.

## Key Points

- Applications of ChatGPT in Medical Research: ChatGPT has shown potential in aiding medical research at the hospital level by offering academic writing assistance, data analytics, statistics handling, and code generation capabilities. It helps streamline tasks, support decision-making, and improve patient interaction while also serving as a valuable tool for preliminary data exploration and simplifying complex datasets.
- Data Security and Patient Confidentiality: A major concern with AI systems like ChatGPT is the handling of sensitive healthcare data on cloud servers, which may pose data privacy and security risks. Ensuring de-identification of data and adherence to data protection regulations like HIPAA or GDPR is crucial.
- Reliability, Biases, and Data Hallucinations: AI systems, including ChatGPT, can produce erroneous or biased outputs based on their training data. There’s also the risk of data hallucinations where the model may generate inaccurate or fabricated information. Ensuring diverse training data and validating AI outputs through established methodologies are essential steps to mitigate these issues.
- AI Interpretability and Induced Disparities: The “black box” nature of AI systems poses challenges in understanding their decision-making processes, which can be problematic in healthcare settings. Additionally, AI systems could perpetuate or exacerbate existing biases if trained on unrepresentative data, leading to disparities in health care.
- Regulatory Frameworks and Future of AI in Medical Research: Regulatory bodies are working towards developing frameworks to guide AI use in health care, addressing challenges posed by AI’s dynamic nature. The future of AI in medical research is promising, with the potential to revolutionize epidemiological studies, diagnostics, and patient care, yet requires a balanced approach to ensure ethical considerations and data security.
